# Development of the Measuring Techniques for Estimating the Air Void System Parameters in Concrete Using 2D Analysis Method

**DOI:** 10.3390/ma13020428

**Published:** 2020-01-16

**Authors:** Agnieszka Molendowska, Jerzy Wawrzeńczyk, Henryk Kowalczyk

**Affiliations:** Faculty of Civil Engineering and Architecture, Kielce University of Technology, Al. Tysiąclecia Państwa Polskiego 7, 25-314 Kielce, Poland; zmsjw@tu.kielce.pl (J.W.); hkowalczyk@tu.kielce.pl (H.K.)

**Keywords:** concrete, air void distribution, image analysis, surface area, Schwartz–Saltykov method

## Abstract

The purpose of the present study was to determine the impact of image quality on the results of air void system parameters determination in air-entrained concretes. The focus was on technical aspects related to the preparation of the scanned image of the concrete surface, which was then subjected to 2D surface analysis. Image processing aimed at separating joined voids and removing various types of defects in aggregate and cement mortar. The specific surface of the voids was determined with the air void equivalent diameter or perimeter as the calculation basis. Applying the Schwartz–Saltykov method, the 3D distribution of the air voids was reconstructed based on 2D measurements. On this basis, the micro-air content A300 was determined. The results of the 2D method were compared with the results of determinations carried out using the linear traverse (1D) method according to EN 480-11. The tests confirm the need to correct the image prior to measurements. Comparative tests showed good agreement between the air void system parameters determined using the 2D analysis and the EN 480-11 chord length counting method.

## 1. Introduction

Air-entrainment of a concrete mix is the basic technological process aimed at ensuring the resistance of moist concrete to the cycles of freezing and thawing. Although this method has been known for over 70 years, the issues related to proper air-entraining and quality control have not been completely resolved. Theoretical and technical issues still discussed include the selection of parameters that better describe the air void system in the context of concrete freeze-thaw resistance assessment, measurement methodology (linear or surface, manual or automated image analysis), and sample preparation (contrasting, extracting air voids from the background).

A spacing factor [1] that is based only on the total void specific surface and volume, without regard to the true number of voids, cannot be a truly definitive parameter. Other researchers have attempted to use the chord distribution curve to represent the air void dispersion. Many other measures for the air void system characterization have been devised (Philleo 1983 [2], Attiogbe 1993 [3], Elsen et al. 1994 [4], Pleau and Pigeon 1996 [5], Hasholt 2013 [6], Mayercsik et al. 2014 [7] Murotani, Igarashi et al. 2019 [8]). None of those methods have been adopted for general use. Methods of determining the Philleo factor from 2D measurements were proposed by Załocha and Kasperkiewicz [9], Wawrzeńczyk and Kozak [10], and by Song et al [11]. Parameters of the air void system have been determined through fractal analysis [12,13] and recently, directly in 3D space, using X-ray computed tomography [14,15,16,17,18]. 

The traditional way of determining the air void characteristics of concrete is by microscopic examination, as described in standards ASTMC 457 or EN 480-11 [19,20]. The most common technique is the linear traverse method which involves optical observations of the polished concrete surface and measurement of the air void chord intercepts. The angle of incidence and intensity of illumination must be appropriately selected [19].

Sample preparation is a critical step in this technique. By grounding and polishing the flat surface of the specimen, details such as air voids, aggregates and cement paste are revealed. 

A traditional lineal analysis of air-entrained concrete is operator-dependent. A human operator observes the specimen through a microscope while it is being traversed and defines air void edges subjectively. Subjective decisions must also be made as to whether an observed void is or is not an air void [21].

The calculations are extremely sensitive to errors made in the determination of the number of voids traversed. The accuracy of a linear traverse determination of the air-void parameters is dependent on the number of voids encountered and measured along the traverse as it is on the length of the traverse. Snyder et al [22] conducted an analytical investigation of the effect of the number of voids and the length of the traverse on the minimum expected error. If the number of voids is very small because of low air content or large voids, the length of traverse recommended in ASTM C 457 is probably insufficient to obtain accurate air-void parameter data [22].

As the conventional manual measurements are tedious, time-consuming, and operator-dependent, semi-automated and automated image analysis (AIA) techniques have been attracting the interest of researchers since the 1980s. Numerous efforts have also been directed towards automating the linear traverse method. 

The naturally occurring contrast between air voids and the surrounding cement paste is insufficient for accurate air void delineation by image analysis equipment. It is therefore necessary to increase this contrast in the procedure of surface preparation. The degree of flatness of the polished specimen surface needs to be higher than that required in the traditional microscopic examination, and the paste edges at the interface of voids, must be sharply defined [23]. As reported by Chatterji and Gudmundsson [24], surface defects such as cracks, crushed aggregate and particles plucked out of the cement paste can make automated identification of concrete specimen constituents more difficult. Considering this fact, the internal voids in the aggregate have to be masked before the examination by painting the aggregate with a permanent ink pen under a stereomicroscope. A typical surface preparation is to ink the surface black, fill the voids with a white powder, remove the excess from the surface, and finally, ink out any filled defects [25]. 

Automated image analysis can be performed in two ways: (a) analysis of many single images of small size or (b) analysis of one large image created after stitching many small images or after scanning the large surface of a concrete sample [11,26,27].

In the first approach, the specimen is analysed using a large number of small, one-size fields of measurement. The field boundaries on the specimen can truncate a significant number of features whose measured or apparent size will be smaller than their true size, leading to underestimations. Since the features with their centres outside the field of view will often be partially imaged, the number of features per unit area will be overestimated. These resulting measurement distortions are referred to as the frame edge effects. The frame edge effect does not affect area percent measurements because individual feature size is not a factor in this determination [23].

In the second approach, a 2D method is used to analyse a large image of the specimen surface. The first benefit of this approach is that it allows measurement of all features present in the field of view. This produces much more information (counts more features or directly measures distances) with less effort. The features such as area, equivalent diameter, circumference, circularity, convexity, shape factor, etc., are determined for each air-void present in the selected region. 

The most widely used automated system based on black and white contrast enhancement, the RapidAir 457, relies on an operator to set the threshold level for each analysis [28].

Recently, more sophisticated methods have been developed that do not require black-and-white contrast enhancement of the lapped section. Dedicated algorithms are used to recognize entrained air voids based on the shadows cast during oblique illumination. 

Song et al. [11] used an approach called spectral analysis which was adopted to segment each scanned RGB image. Polished concrete surfaces were treated with phenolphthalein and a florescent chalk powder to distinguish between the phases. Free open-source software Multispec was employed to segment air void, aggregate, and paste phases [29]. The HF-MAC01 device [30] detects differences in shadowed areas when a specimen is subjected to light at various angles. One of the NG-ACE analysis approaches [31] is colour recognition using neural networks pre-trained to identify the specific colour features of each component. This produces a colour map of the original image which is correlated with the identified components. Neural networks for detecting and classifying air voids are also employed by the newer version of the Nikon Imaging System (NIS).

Modern air-entrained concretes have a much more complex air void system than those in the 1950s and 1960s. Observed voids often do not have the expected spherical shape. They are also smaller than those obtained with air entraining admixtures used previously and may occur in clusters. This is a departure from the premise of the standard method of the spacing factor determination. Errors in the evaluation of this parameter may consequently affect the assessment of concrete freeze-thaw resistance.

The present study was focused on the technical aspects related to the preparation of a large scanned image of concrete surface, which was then subjected to the 2D analysis. The purpose was to determine the impact of image quality on the determination of air void system parameters in air-entrained concretes. The results of the 2D method were compared with the results of determinations carried out using the linear traverse (1D) method according to EN 480-11.

## 2. Materials and Methods 

It is difficult to design concrete with specific parameters of the air void system. From the perspective of the test methodology, we assumed the specific surface area α to be the basis for the differentiation. In the course of routine tests of surface concretes (EN-480-11), we selected 5 concretes differing in the parameter α. The tested concrete samples of class C35/45 with w/c = 0.37–0.39 were made with CEM I 42.5 cement and granite aggregate with a maximum particle size of 22.5 mm.

Two types of tests were carried out, the standard EN 480-11 test and the 2D analysis method. First, the standard tests [20] were performed to select 5 concretes with different void specific surface area α (the basis for spacing factor determination). The air void system in the tested concretes was not typical due to the high content of very small air voids.

For each concrete, two 100 mm × 150 mm× 40 mm specimens were prepared. The test surfaces were ground by the wet process and polished as per EN 480-11.

### 2.1. EN 480-11 Method

**Figure 1:**
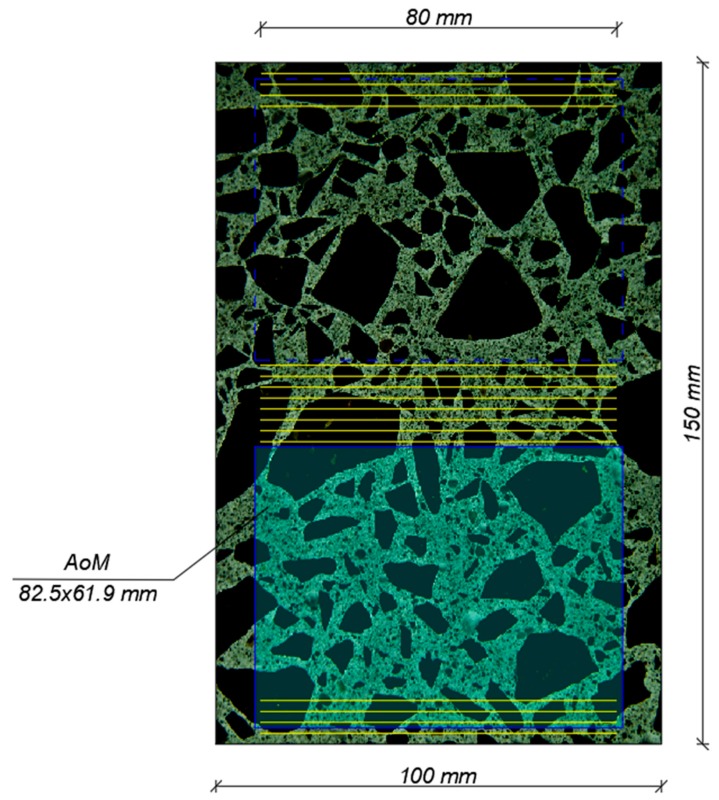
The arrangement of 16 traverse lines (1D) and area of measurement (2D).

The procedures were applied in accordance with the EN 480-11 standard to two 100 mm × 150 mm × 40 mm specimens. The specimen natural surfaces were examined. The air voids were made distinguishable with side lighting (according to the lighting procedure described in ASTM 457). The arrangement of 16 traverse lines is shown in Figure 1. The scanning hardware used is described in the next subsection. A series of images were captured while the stage-mounted specimen was moving along the traverse line. A total of 28 frames (1280 × 960 pixels with a resolution of 1.13 μm/px) were tiled to form one large colour image, about 80 mm in length. The NIS-Elements program was used to further analyse the output image. A traverse line was applied along the image and the chord lengths were measured manually using the NIS-Elements tool. The NIS-Elements program records individual chord lengths and allows exporting the recorded data to a spreadsheet (MS Excel). When the input data such as the paste volume (P) and the total traverse length (Ttot) are added, the prepared VBA macro completes calculations according to EN 480-11.

Unlike ASTM 457, the EN 480-11 standard includes the procedure that can be used to determine the air void distribution in 3D space. The result of the counting procedure is a distribution table as per EN 480-11. All the recorded chords are subdivided into 28 classes of different lengths from 0 to 4000 μm. The micro-air content A_300_, expressed as percent by volume, is read directly from the distribution table as the sum of the volume attributed to all air voids with diameters from 0 to 300 μm (classes 1–18). The values obtained from the calculations will also include the basic air void system characteristics: total air content, (A), spacing factor, ($\bar{L}$), and air void specific surface area (α).

The calculated distribution is based on the model which assumes only a nominal set of air void diameters that correspond to the maximum chord length in each of the classes. It is worth noting that in this method, not every void within the cement paste will have been intersected during the traverse, as the traverse lines do not cover the whole volume of the concrete specimen.

### 2.2. 2D Analysis

When the standard measurements were completed, the surfaces were prepared for 2D analysis. The specimens were contrast enhanced to distinguish individual concrete phases. A few attempts were made to select the most effective way to prepare the polished surfaces for analysis, such as painting the surface with a black permanent marker or applying acrylic ink. Ultimately, covering the specimen with a blue permanent marker was found to be the best option. In addition, the surface of the specimen was coated with a well-penetrating paste (used in furniture care) to protect the dye against abrasion while filling the voids with powder. Finally, the air voids were filled with white powder. After trying barium sulphate and zinc oxide, zinc oxide was chosen as the better option. 

Automated image analysis was carried out using a professional set which included a stereoscopic microscope (Nikon SMZ1500), a CCD camera (Nikon DS-Fi1), an automated x-y stage (Prior ProScan III), and a computer with NIS-Elements image analysis software (version BR). For the 2D measurements, a system making use of a ring light was applied.

For each specimen, one image was prepared, consisting of 10 × 10 frames of 1280 × 960 pixels, with a resolution of 6.75 μm/px. The combined colour image had dimensions 82.5 mm × 61.9 mm (Figure 1). For colour images, the NIS-Elements program automatically selects the threshold value at which the air void phase (white circles) is isolated on a much darker background. It was about 165 for the image under analysis. As a result of thresholding, binary images often need some modifications before they can be used for measurements. The void phase appeared bright on the dark blue background. The blue marker pen made the particles of sand, coarse aggregate, and the paste clearly visible, which contributed to easier image correction. The quality of the combined image is of key importance in 2D analysis. Manual correction needs to be performed, which involves removing various defects from the image, such as scratches, cracks, voids in the aggregate and in the paste-aggregate interfacial zone, and merged and deformed air voids in the paste. During the measurements, the shape of the void should be properly reconstructed [20]. This is a very tedious job but necessary for the quality and accuracy of calculations. Such a correction is necessary in both 1D and 2D image analysis techniques. In addition to drawing tools (NIS-Elements image analysis software), the image processing tools available in the program include mathematical morphology commands such as clean, fill holes, close, and open. The image preparation stage is followed by calculations performed using the automatic measurement tool. The parameters recorded for each air void in the NIS-Elements program were: Area (Area), Equivalent Diameter (EqD), Perimeter (Perim), Elongation and Circularity. The measured values were exported to MS Excel, where further calculations were performed using the VBA macro.

**Figure 2:**
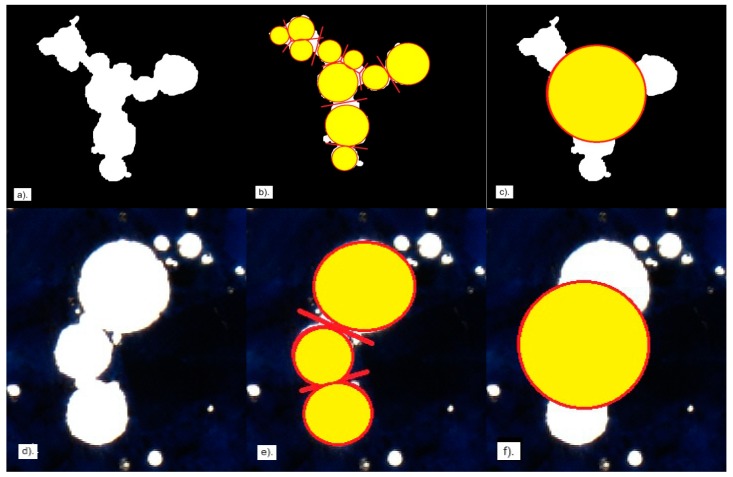
Examples of air void grouping (**a**,**d**) connected air voids, (**b**,**e**) breakup of agglomerated air voids, (**c**,**f**) an individual air void having equivalent diameter.

Figure 2 shows examples of adjoining air voids in the specimens that were analysed. The method of separating the voids is of key importance for obtaining the correct calculation results. As recommended by Pleau et al. [32], two connected air voids were treated as a single unit when the centre of the smaller void was inside the larger void. Otherwise, the air voids were treated as two individual voids.

The basis for calculating the spacing factor (L) is the void-specific surface, which is determined from the area (Area), equivalent diameter (EqD) or perimeter (Perim) of individual voids. Both the equivalent diameter and the perimeter change as a result of separation, so does the value of parameter α. After separation, several single air voids are distinguished that have smaller diameters but increased cumulative perimeter (Figure 2b,e). A set of voids treated as one unit gives a larger equivalent diameter and a smaller perimeter (Figure 2c,f).

The NIS-Elements program separates the voids automatically or manually, making use of image editing tools.

Three variants of air void separation were adopted in the analysis:

- no separation,
- automatic separation,
- manual separation.

The automatic air void separation option is available in the NIS program but in the case of large and irregular voids, the algorithm does not work correctly—sometimes a void becomes separated into several parts. For this reason, large voids were treated in two ways: i / all voids included in the sample were taken into account and ii / voids more than 2 mm in diameter were excluded. Manual air void separation is effective but very laborious. 

The specific surface α in the 2D method was calculated in two ways, using the equivalent diameter EqD (Equation (1)) or the perimeter Perim (Equation (2)) as the basis for calculations [4]:

(1) $$\alpha_{1}=\frac{4}{3}\pi \cdot \frac{\sum \mathrm{EqD}}{\sum \mathrm{Area}}$$

(2) $$\alpha_{2}=\frac{4}{\pi }\cdot \frac{\sum \mathrm{Perim}}{\sum \mathrm{Area}}$$

The air content in the concrete was determined as the ratio of the sum of air void surface area to surface area of the specimen being analysed (Equation (3)).

(3) $$A=\frac{\sum \mathrm{Area}}{\mathrm{AOM}}$$

where AOM represents the area of measurement;

If P/A > 4.342 (P—volume of cement paste), then $\bar{L}$ is calculated from

(4) $$\bar{L}=\frac{3[1.4{\left(1+R\right)}^{1/3}-1}{\alpha }$$

where: R—is the paste/air ratio

If P/A ≤ 4.342, then $\bar{L}$ is calculated from

(5) $$\bar{L}=\frac{P}{A\cdot \alpha }$$

### 2.3. Determining Micro-Air Content A_300_

It is not possible to determine the exact micro air content A_300_ in a volume unit using the 2D method; therefore, only the estimated value is determined. The air void distribution in 3D is determined based on the known distribution of measured void diameters on a 2D surface. In this work, only air voids up to 300 μm in diameter were considered. Calculations were performed assuming 15 classes of width Δ = 20 μm for both 2D and 3D distributions.

A section in the i class appears in two cases, when a sphere with diameter d(i) is cut by the plane on its diameter plane or when it is cut on a different plane. The probability that a void with diameter in the j class produces a section with a diameter in the i class can be expressed as [33,34]

(6) $$B_{\mathrm{ij}}=\{\begin{array}{ll} \sqrt{j^{2}-{\left(i-1\right)}^{2}}-\sqrt{j^{2}-i^{2}},\; & \mathrm{if}\;i\leq j\\ 0, & \mathrm{if}\;i<j \end{array}$$

Therefore, the procedure of counting air void per unit volume in a class j (N_v_(j)) from the number of voids in the unit area across the classes (N_a_(j)) has to involve subtracting the number of spheres in the bigger classes multiplied by the probability that they have produced a section in the j class.

An estimate of the number of air voids per unit volume in each class N_v_(j) can be calculated from [33,34]:

(7) $$N_{v}\left(j\right)=\frac{1}{\Delta }\sum_{i=j}^{N}\alpha_{\mathrm{ij}}N_{a}\left(i\right)\;\mathrm{for}\;j=1\ldots N$$

The matrix of coefficients α_ij_ (reciprocal of B_ij_) is determined from

(8) $$\alpha_{\mathrm{ij}}=\left\{ \;\begin{array}{ll} T_{\mathrm{ii}},\; & \mathrm{if}\;i=j\\ -\sum_{m=i}^{j-1}\alpha_{\mathrm{mi}}T_{\mathrm{mj}},\; & \mathrm{if}\;i<j \end{array}\right.$$

where T_ij_ is defined as

(9) $$T_{\mathrm{ij}}=\{\;\begin{array}{ll} \frac{1}{B_{\mathrm{jj}}},\; & \mathrm{if}\;i=j\\ \frac{B_{\mathrm{ij}}}{B_{\mathrm{jj}}},\; & \mathrm{if}\;i<j \end{array}$$

The air content as a function of air void diameters is calculated as follows [35]

(10) $$A_{3D}=\frac{\pi }{6}\sum_{i=1}^{N}N_{v}\left(i\right)d{\left(i\right)}^{3}$$

where N_v_(i) are the counts per volume and d(i) represents the mean air void diameter in the i-th class.

According to the standard method, the air volume is determined assuming that all air voids in a given class have a diameter equal to the upper limit of the class. This can be correct when not many chords are recorded in individual classes. The adoption of such a formula in the 2D method will lead to a significant overestimation of the air void volume as in some classes, from several to several thousand voids are recorded. The volume of a single air void in a given class V_1_ is calculated by adopting d_m_(i), i.e., the average diameter, which is close to the arithmetic average of the diameters in the given class. The void volume in the given class is calculated according to the formula: V_3D_(i) = N_v_(i)*V_1_(i). In the next step, the micro air content by diameter is determined by adding up V_3D_ in individual classes.

**Figure 3:**
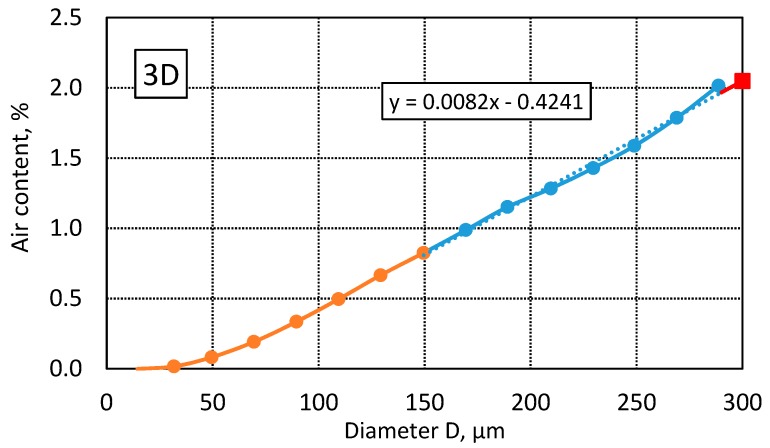
Method of determining micro-air content A_300_.

Since average diameters in the classes are considered in the calculations, it is necessary approximate the value for d = 300 μm to determine the micro-air content A_300_. The result is obtained by calculating the linear function coefficients for the results in the diameter range 150–290 μm. The calculation principle is shown in Figure 3.

A VBA macro prepared in MS Excel was used to make all the calculations and the results were saved in a text file. Example calculation results are shown in Table 1. To perform the calculations, the input data such as the image size (B, H) and resolution (PX) must be entered along with the information about the paste content (P). The program calculates the number of air voids (N) and air content (A), and two variants of specific surface (α_1_, α_2_) and spacing factor (L_1_, L_2_), depending on whether the equivalent diameter (EqD) or perimeter (PERIM) is used in the calculations. The results include the number (ni) of void diameters in each class (15 classes in the 0–300 µm range), the mean air void diameter in a given class (dm(i)), the number of air voids per unit area (N_a_(i)), the number of air voids per unit volume (N_v_(i)), and the total air void volume (Sum_V3). The micro-air content A_300_ is determined from Sum_V3 calculations, as shown in Figure 3.

When the number of recorded air voids was too large (exceeded 32,000), MS Excel could not complete the calculations. Therefore, some of the calculations were performed with Statistica software, which does not have such limitations.

## 3. Results and Discussion

The air void system was characterized using two methods:

- standard method to EN 480-11 (Table 2),
- 2D image analysis, with three variants of result presentation:
  – leaving the connected air voids unseparated—2D (Table 3),
  – separating the connected air voids automatically in NIS-Elements—2Da (Table 4),
  – separating the air voids manually—2Dm (Table 5).

In each of the variants, the results were presented for all air voids, considering only those up to 2 mm in diameter (values in parentheses). In the 2D analysis with automatic air void separation, for the air void, up to 2 mm system parameters were determined in two ways: by rejecting the air voids with diameters of more than 2 mm before the separation (first approach) or after the separation (second approach). Since no significant differences were observed between the results of both approaches, only those from the second approach are presented.

Accurate verification of the results would be possible if the standard model concrete with known exact void distribution in 3D space was available for comparison or if air void spacing results from CT scans could be obtained. Since we did not have a CT scanner, we compared the results of our 2D analysis with the standard procedure results. 

Data spread assessment was the basis of the analysis. We are aware that at this stage of the research, we have no reference/benchmark to use for comparison. The analysis consisted in comparing the results obtained from the standard method tests with the results obtained using the 2D method. We assessed how convergent the results were in terms of equivalent diameter or perimeter and various ways of separating the interconnected air voids.

Agreement between the results obtained from the 2D and standard methods was assessed by determining the ν_k_ coefficient (Equation (11))—the root mean square relative deviation of 2D results (y) with respect to the results from the procedure set forth in EN 480-11 (Y) (Table 6):

(11) $$\nu_{k}=100\sqrt{\frac{\sum {\left(\frac{Y-y}{Y}\right)}^{2}}{n-1}}$$

**Figure 4:**
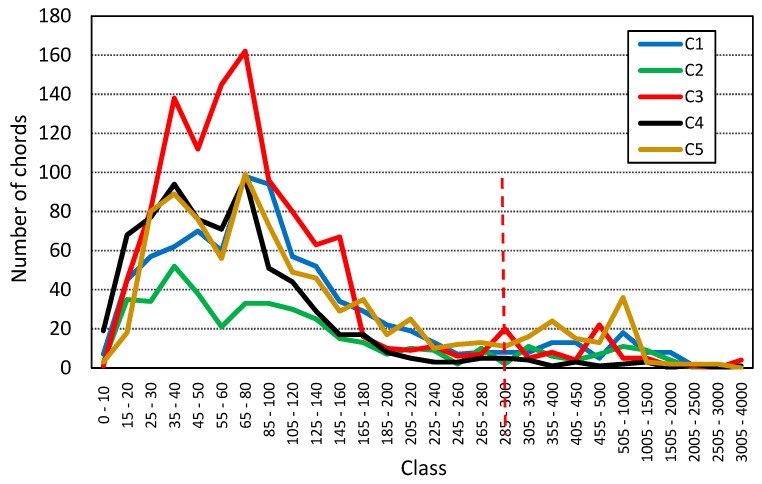
Chord length distribution by class for C1–C5 concretes.

The chord length values are presented in Figure 4 and the calculated values of standard parameters are compiled in Table 2. Table 3, Table 4 and Table 5 summarize the 2D analysis results in terms of different procedures of connected air void separation.

As indicated in Figure 4, most chords are in the range up to 300 µm. Thus, only the air voids that were up to 300 µm in diameter were determined in the air void distribution procedure.

**Figure 5:**
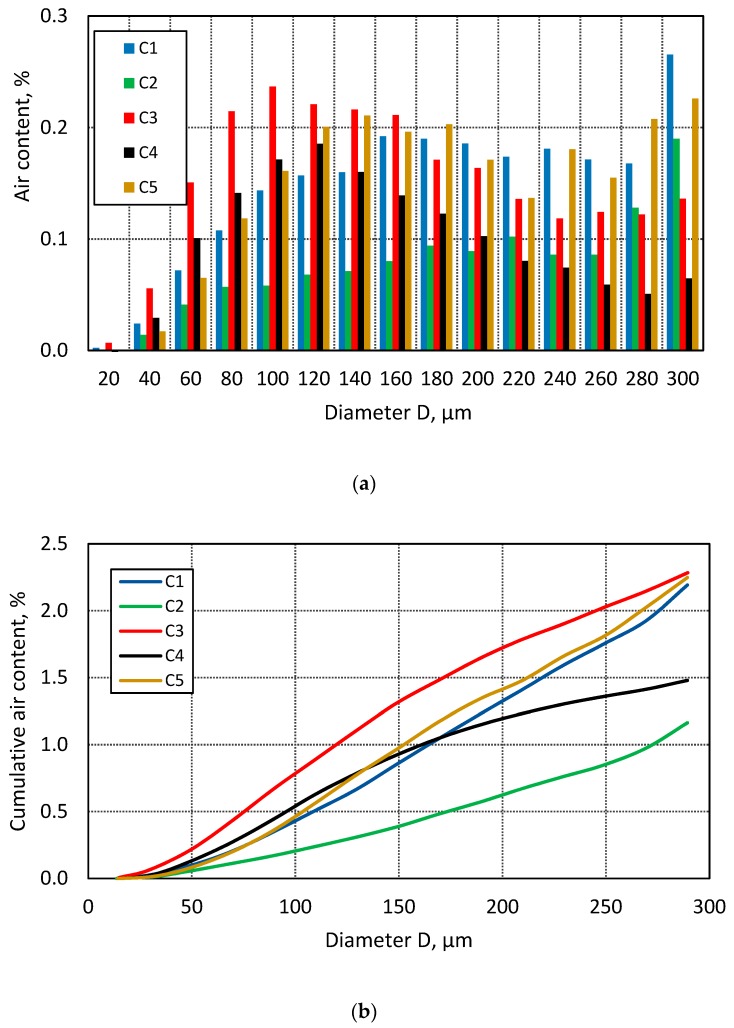
(**a**) Air content in a given class, (**b**) cumulative air void volume for concretes C1–C5 (2D m).

The histogram in Figure 5a compares the air content in each of the classes. Figure 5b illustrates the distribution of the cumulative air content in terms of the void diameter. 

The image of the concrete specimen needs to be carefully prepared before analysis and calculations. Elimination of defects occurring in the digital image (scratches, cracks, paste-aggregate interface artefacts) is tedious but essential. Because the granite aggregate used in the concrete had numerous cracks, image processing required a lot of work. Better correlation of the EN 480-11-based results with the 2D method-based results was achieved when the connected air voids were separated. Table 5 shows that after the separation, the ν_k_ values are usually a few percents lower. Air voids can be separated in various ways, using the automated separation option available in the computer program (2D a) or manually, using image editing tools (2D m). The automated separation (2D a) applied together with a slight manual correction, which additionally shortens the time of image preparation was regarded as the most beneficial variant. 

**Figure 6:**
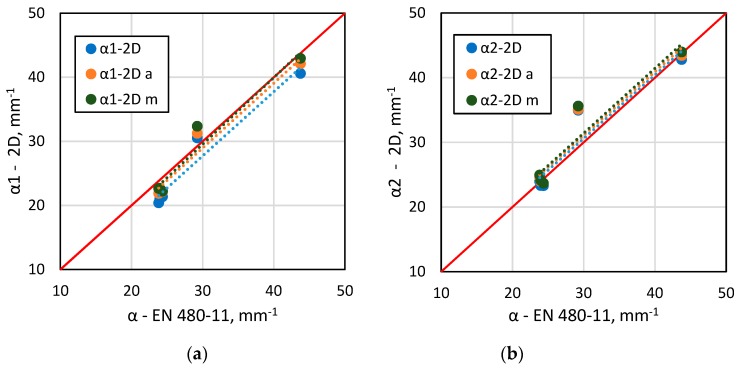
A comparison of EN 480-11 data versus 2D analysis results for α: (**a**) equivalent diameter based 2D analysis; (**b**) perimeter based 2D analysis.

**Figure 7:**
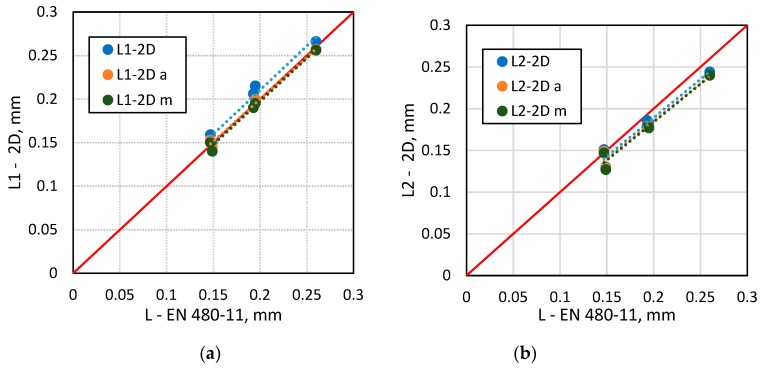
A comparison of EN 480-11 data versus the 2D analysis results for $\bar{L}$: (**a**) equivalent diameter based 2D analysis; (**b**) perimeter based 2D analysis.

**Figure 8:**
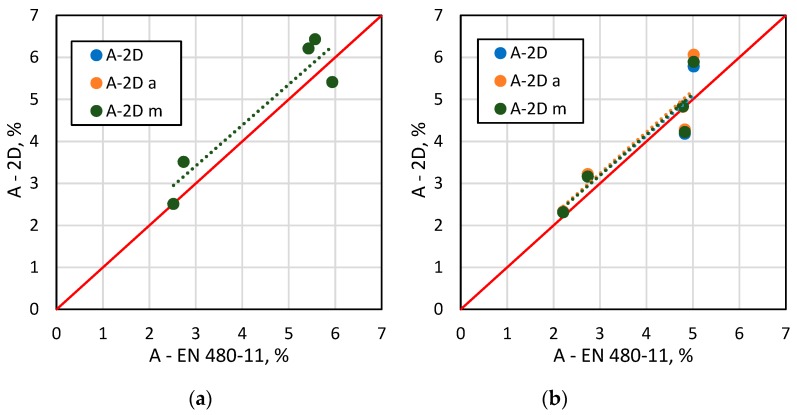
A comparison of EN 480-11 data versus the 2D analysis results for A: (**a**) all air voids, (**b**) air voids up to 2 mm in diameter.

**Figure 9:**
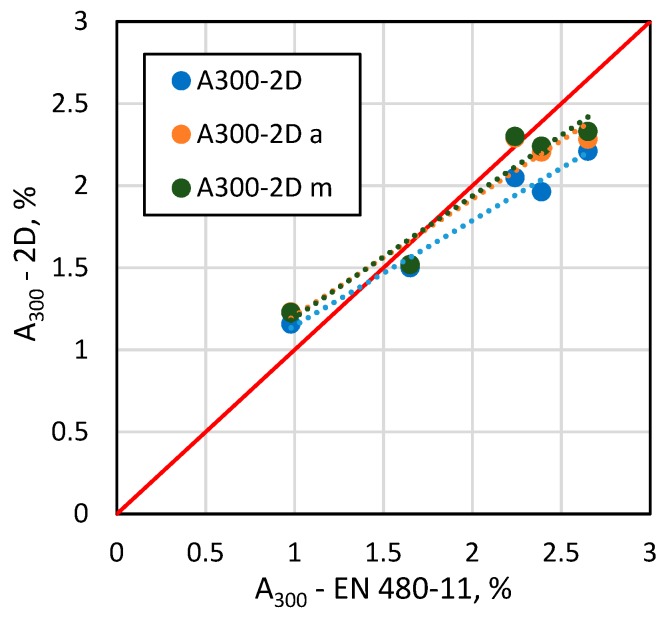
A comparison of EN 480-11 data versus the 2D analysis results for A_300_.

Figure 6, Figure 7, Figure 8 and Figure 9 show selected results of air-void system parameters obtained by various methods. The comparison of EN 480-11 based parameter values with the 2D based values shows good agreement between the results (Table 6). In the case of the specific surface α and spacing factor $\bar{L}$, the standard and 2D determinations were in better agreement when all the air voids occurring within the test area were included in the analysis (Table 6). In contrast, for air content A, the results were better correlated when only air voids up to 2 mm in diameter (ν_k_ = 13–15%) were included (Figure 8 and Table 5). Considering that in the standard method, the surface of the specimen is analysed only along the test lines and in the 2D method, only a selected area is examined, leaving out a few large air voids in the analysis will affect the results.

In the 2D method, the specific surface of air voids α was calculated by adopting the equivalent diameter (α_1_) or perimeter (α_2_) as the basis for calculations. Better agreement between the specific surface α (ν_k_ = 8%) results and the $\bar{L}$ spacing factor (ν_k_ = 3%) was found when the equivalent diameter was used (Figure 6 and Figure 7 and Table 6). Calculations based on the air void perimeters yielded worse results represented by ν_k_ = 10–11% and ν_k_ = 10% for the α and $\bar{L}$ parameters, respectively. These findings vary from those obtained by Fonseca and Scherer [26].

A satisfactory correlation was found between the micro-air content A_300_ results from the standard method EN 480-11 and those based on the Schwartz–Saltykov method (ν_k_ = 15%) (Figure 9 and Table 6).

The deviation mainly relates to the A_300_ parameter with higher values around 2.5% determined according to EN 480-11. It can be assumed that in the standard method, some large air voids (more than 300 µm in diameter) can give chords that are classified in the range up to 300 µm, resulting in an increased micro-air content A_300_.

We are convinced that 3D analysis of air void distribution based on measurements of void diameters (EqD) on a 2D surface is more reliable compared to chord measurements. The same chord length can be obtained by cutting a small or large void, which is impossible when diameters are measured.

## 4. Conclusions

The results of this study confirm the observations of other authors about the importance of careful preparation of concrete samples for automatic image analysis. A 2D analysis specimen must be polished much more carefully than a 1D linear analysis specimen. Additional image processing is often necessary. 

The 2D automated method is relatively simple when dealing with the air entrained concrete in which the air voids are isolated and circular with relatively large diameters. Modern air-entrained concretes have a much more complex air void system than those produced in the 1950s and 1960s. The use of various additives and combinations of chemical admixtures in concrete nowadays raises new challenges, as do concrete mixes with higher liquidity. As a result, the air void shapes often deviate from the expected sphere, are smaller than those obtained with previously used air entraining admixtures, and may occur in clusters. The shape deviations, in addition to measurement variations, produce various errors in the analysis. 

In our view, the 2D method provides a better evaluation of actual air void parameters than the linear travers method (1D). The primary benefit of 2D image analysis is that it is capable of measuring all the features present in the field of view, thereby producing much more information with less effort. Each air void present in the selected region is evaluated in terms of specific area, equivalent diameter, circumference, circularity, convexity, shape factor, etc. The 2D specimens of concrete or mortar can be smaller than the standard ones (100 mm × 150 mm as per EN 480-11). 

Due to the complex air void arrangement, the concrete specimens in this study were subjected to additional operations. The results obtained lead us to the conclusion that automated separation of combined voids with the use of morphological functions available in the computer program plus manual separation of the voids is the optimum solution. Comparison of the 1D and 2D results show the better match when the 2D analysis adopts equivalent diameters of the voids as the basis for calculation. 

The automated 2D image analysis method requires further development of algorithms to facilitate the capture of combined air voids, their clusters, and their separation.

The results did not define clearly which method is better. Accurate verification of the results obtained would be possible if we had a concrete pattern with known exact void distribution in 3D space. Then, making 2D measurements could compare the measurement results to the actual distribution.

Another option is to compare the conversion results from 2D to 3D measurements with the void distributions obtained from computer tomography. In the near future, we will have this opportunity. We hope that the continuation of research in this direction will give a more reliable assessment of the effectiveness of the automated method. 

## Figures and Tables

**Table 1:** Results of calculations with the use of the VBA macro in MS Excel.

Concrete C5–2D						
Area of measurement						
Width B = 12224 (x)						
High H = 9168 (px)						
Calibration PX = 6.7704 (mm^−3^/px)						
Area AOM = 10274.1664 (mm^2^)						
Volume of cement paste P = 27.7 (%)						
Number of pores N = 31599						
Air content A = 6.43 (%)						
Calculation based on equivalent diameter (EqD)						
Specific surface α_1_ = 21.00 (mm^−1^)						
Spacing factor L_1_ = 0.206 (mm)						
Calculation based on perimeter (PERIM)						
Specific surface α_2_ = 23.31 (mm^−1^)						
Spacing factor L_2_ = 0.186 (mm)						
No	Class	ni	N_a_(i)	dm(i)	N_v_(i)	Sum_V3
1	0-20	153	0.015	14.487	10.008	-0.001
2	20-40	6079	0.592	31.731	17.619	0.017
3	40-60	6719	0.654	49.519	10.997	0.082
4	60-80	4961	0.483	69.479	6.103	0.192
5	80-100	3499	0.341	89.535	3.667	0.335
6	100-120	2453	0.239	109.407	2.238	0.496
7	120-140	1735	0.169	129.238	1.171	0.666
8	140-160	1197	0.117	149.534	1.094	0.825
9	160-180	905	0.088	169.483	0.669	0.988
10	180-200	684	0.067	189.116	0.522	1.152
11	200-220	481	0.047	209.588	0.314	1.283
12	220-240	408	0.040	229.608	0.284	1.429
13	240-260	339	0.033	249.006	0.184	1.587
14	260-280	288	0.028	269.003	0.153	1.787
15	280-300	201	0.020	288.702	0.171	2.016
Micro air content A_300_ = 2.049%						

**Table 2:** Results of the air void parameter assessment as per EN 480-11.

Series	P %	EN 480-11				
		A %	A_300_ %	N	α mm^−1^	$\bar{L}$ mm
C1	27.4	5.43 (4.79)	2.39	820 (815)	23.83 (26.86)	0.20 (0.18)
C2	27.4	2.74 (-)	0.98	424 (-)	24.36 (-)	0.26 (-)
C3	26.0	5.94 (4.83)	2.65	1124 (1118)	29.25 (35.88)	0.15 (0.13)
C4	26.0	2.52 (2.21)	1.65	706 (703)	43.76 (49.67)	0.15 (0.14)
C5	27.7	5.57 (5.02)	2.24	854 (849)	23.98 (26.53)	0.19 (0.18)

Numbers in parentheses indicate the values for the air voids up to 2 mm in diameter. (-) no air voids with more than 2 mm in diameter.

**Table 3:** Results of the air void parameter assessment with 2D procedure.

Series	2D						
	A %	A_300_ %	N	α_1_ mm^−1^	${\bar{L}}_{1}$ mm	α_2_ mm^−1^	${\bar{L}}_{2}$ mm
C1	6.21 (4.83)	1.96	36013 (35995)	20.39 (25.75)	0.22 (0.19)	23.94 (30.07)	0.18 (0.16)
C2	3.51 (3.16)	1.16	17802 (17797)	21.33 (23.47)	0.27 (0.25)	23.29 (25.59)	0.24 (0.23)
C3	5.41 (4.18)	2.21	66009 (65998)	30.52 (39.14)	0.15 (0.13)	34.94 (44.67)	0.13 (0.12)
C4	2.51 (2.33)	1.50	34055 (34053)	40.57 (43.58)	0.16 (0.15)	42.79 (45.95)	0.15 (1.15)
C5	6.43 (5.78)	2.05	31599 (31589)	21.00 (23.16)	0.21 (0.20)	23.31 (25.60)	0.19 (0.18)

Numbers in parentheses indicate the values for the air voids up to 2 mm in diameter.

**Table 4:** Results of air void parameter assessment with 2D with automatic separation of connected air voids.

Series	2D a						
	A %	A_300_ %	N	α_1_ mm^−1^	${\bar{L}}_{1}$ mm	α_2_ mm^−1^	${\bar{L}}_{2}$ mm
C1	6.21 (4.82)	2.21	38212 (38185)	21.86 (27.59)	0.20 (0.18)	24.58 (30.80)	0.18 (0.16)
C2	3.51 (3.22)	1.23	18502 (18497)	22.12 (23.90)	0.26 (0.25)	23.66 (25.54)	0.24 (0.23)
C3	5.41 (4.28)	2.28	67614 (67601)	31.22 (39.49)	0.15 (0.13)	35.10 (44.27)	0.13 (0.12)
C4	2.51 (2.33)	1.52	35367 (35364)	42.08 (45.14)	0.15 (0.15)	43.40 (46.54)	0.15 (1.14)
C5	6.43 (6.06)	2.29	34214 (34206)	22.63 (23.97)	0.19 (0.19)	23.93 (25.34)	0.18 (0.18)

Numbers in parentheses indicate the values for the air voids up to 2 mm in diameter.

**Table 5:** Results of air void parameter assessment by 2D with manual separation of connected air voids.

Series	2D m						
	A %	A_300_ %	N	α_1_ mm^−1^	${\bar{L}}_{1}$ mm	α_2_ mm^−1^	${\bar{L}}_{2}$ mm
C1	6.21 (4.83)	2.24	39272 (39255)	22.61 (28.03)	0.20 (0.18)	24.94 (30.82)	0.18 (0.16)
C2	3.51 (3.16)	1.23	18749 (18744)	22.18 (24.41)	0.26 (0.24)	23.66 (26.01)	0.24 (0.23)
C3	5.41 (4.22)	2.33	70090 (70079)	32.34 (41.28)	0.14 (0.12)	35.60 (45.34)	0.13 (0.11)
C4	2.51 (2.31)	1.52	36453 (36451)	42.94 (46.15)	0.15 (0.15)	43.95 (47.23)	0.15 (1.14)
C5	6.43 (5.89)	2.30	34302 (34291)	22.65 (24.58)	0.19 (0.18)	24.11 (26.11)	0.18 (0.17)

Numbers in parentheses indicate the values for the air voids up to 2 mm in diameter.

**Table 6:** Consistency of the results obtained by the given method with the standard-based results—root mean square relative deviation of all the results.

Parameter	ν_k_, %		
	2D	2D a	2D m
$\bar{L}$_1_	7.46 (7.34)	2.92 (5.30)	3.37 (6.07)
$\bar{L}$_2_	8.05 (10.76)	9.62 (11.38)	10.28 (12.27)
α_1_	12.14 (10.30)	7.82 (8.49)	7.94 (9.35)
α_2_	10.13 (14.46)	10.23 (14.54)	11.20 (15.69)
A_300_	16.42	15.62	14.91
A	18.13 (12.99)	18.13 (14.96)	18.13 (13.38)

The numbers in parentheses indicate the results for the air voids up to 2 mm in diameter.
